# Vitamin D Supplementation in Granulomatous Disease: Why Individualized Assessment May Be Safer Than Universal Thresholds

**DOI:** 10.7759/cureus.116660

**Published:** 2026-09-21

**Authors:** Sadia Nadeem

**Affiliations:** 1 General Practice, Punjab Medical College, Faisalabad, PAK

**Keywords:** 1 25 dihydroxy cholecalciferol, 25-hydroxyvitamin d, granulomatous disease, hypercalcemia, hypercalciuria, sarcoidosis, vitamin d metabolism, vitamin d supplementation

## Abstract

Vitamin D deficiency is commonly identified and treated according to serum 25-hydroxyvitamin D (25(OH)D) concentrations. In granulomatous disease, however, this approach may be insufficient because activated macrophages within granulomatous tissue convert 25(OH)D to the active metabolite 1,25-dihydroxyvitamin D (1,25(OH)_2_D) through extrarenal 1alpha-hydroxylase activity. A patient may therefore have a low 25(OH)D concentration while simultaneously having preserved or increased active vitamin D activity, creating a risk of hypercalcemia or hypercalciuria when vitamin D is supplemented. This editorial argues that vitamin D replacement in granulomatous disease should be individualized rather than governed by a universal supplementation limit. Serum 1,25(OH)_2_D should be an important component of the assessment, but it should not be used as a solitary treatment threshold. Serum calcium, renal function, urinary calcium when clinically appropriate, the activity and phenotype of the granulomatous disease, and the indication for vitamin D replacement should also be considered. Current evidence and the American Thoracic Society guideline support assessment of both vitamin D metabolites when replacement is being considered in sarcoidosis, while acknowledging limitations in the evidence base. An individualized approach may better balance the potential skeletal benefits of correcting vitamin D deficiency against the risk of vitamin D-mediated hypercalcemia. Prospective studies are needed to determine whether metabolite-guided supplementation can improve clinical outcomes.

## Editorial

Vitamin D supplementation is routinely used when serum 25-hydroxyvitamin D (25(OH)D) is low. For most patients, this provides a practical framework for correcting deficiency. Granulomatous disease presents a different physiologic situation. Activated macrophages in granulomatous tissue can express extrarenal 1alpha-hydroxylase, increasing conversion of 25(OH)D to 1,25-dihydroxyvitamin D (1,25(OH)_2_D). Increased active vitamin D activity can enhance intestinal calcium absorption and contribute to hypercalcemia and hypercalciuria [1].

This biology creates an important clinical paradox. A patient can have a low 25(OH)D concentration without having a simple deficiency of biologically active vitamin D [1]. Consequently, increasing vitamin D intake solely to normalize 25(OH)D may be inappropriate for some patients with granulomatous disease [1,2]. Severe examples have been reported. In a series of five patients with granulomatous disorders, high-dose vitamin D supplementation preceded hypercalcemic crisis; all patients had parathyroid hormone-independent hypercalcemia and elevated 1,25(OH)_2_D [2]. Such reports highlight the potential hazard of applying conventional supplementation strategies without considering the underlying disease.

The concern is not limited to rare hypercalcemic crises. In a randomized controlled trial involving 27 normocalcemic patients with sarcoidosis and low 25(OH)D, vitamin D supplementation did not improve the measured skeletal surrogate outcomes, and one participant developed significant hypercalcemia during treatment [3]. Observational data have also raised concern about incomplete biochemical assessment before vitamin D prescribing in sarcoidosis. In one study, patients prescribed vitamin D had more frequent hypercalcemia than patients who were not prescribed vitamin D, although the observational design prevents a conclusion that supplementation itself caused all of the observed events [4]. These findings do not justify withholding vitamin D from every patient with granulomatous disease. Instead, they argue against treating a single laboratory concentration in isolation.

A more clinically useful question is therefore not simply, "How low is the 25(OH)D concentration?" but "Is vitamin D replacement appropriate and safe for this individual patient?" The American Thoracic Society clinical practice guideline for sarcoidosis provides an important basis for this approach. When vitamin D replacement is being considered, the guideline recommends assessment of both 25(OH)D and 1,25(OH)_2_D, and it emphasizes close monitoring for hypercalcemia and/or hypercalciuria because the effects of calcium and vitamin D supplementation can be complex [5]. This recommendation recognizes that 25(OH)D alone may not adequately describe vitamin D physiology in sarcoidosis.

In our view, this principle deserves broader consideration across granulomatous diseases. Rather than setting a universal vitamin D supplementation limit, clinicians should individualize the decision using the patient's vitamin D metabolites and calcium homeostasis. Serum 1,25(OH)_2_D is particularly relevant because dysregulated extrarenal production of this active metabolite is a recognized mechanism of granulomatous-disease-associated hypercalcemia [1]. However, 1,25(OH)_2_D should not be treated as an absolute stand-alone dosing threshold. The clinical meaning of a measured value depends on the patient's calcium concentration, renal function, disease activity, and reason for supplementation [1,5].

A physiology-informed assessment could instead consider several variables before supplementation: 25(OH)D, 1,25(OH)_2_D, serum calcium, renal function, and, when clinically appropriate, urinary calcium. The indication for supplementation should also be explicit. A patient with low 25(OH)D, normal calcium, preserved renal function, and no biochemical suggestion of excessive active vitamin D production may have a different risk-benefit profile from a patient with hypercalcemia, hypercalciuria, renal impairment, or evidence of increased active vitamin D activity. Dose, frequency, and follow-up should therefore be individualized rather than automatically escalated until a predefined 25(OH)D target is reached [1,5].

Such an approach does not imply that every patient with granulomatous disease requires repeated 1,25(OH)_2_D testing or that vitamin D supplementation should be prohibited. The evidence remains limited, and the clinical evidence discussed in this editorial is predominantly derived from patients with sarcoidosis rather than other granulomatous diseases. Therefore, extrapolation of these findings to other granulomatous diseases should be approached cautiously. The available randomized trial was small [3], and observational evidence is vulnerable to confounding [4]. There is no validated 1,25(OH)_2_D concentration that independently defines a universally safe vitamin D dose [3-5]. These limitations should temper any recommendation that relies on a single biochemical threshold.

The objective should instead be a shift from threshold-based replacement toward individualized, physiology-informed replacement. Vitamin D deficiency may have consequences for skeletal health, and patients with granulomatous disease may have additional reasons to consider supplementation, including glucocorticoid exposure [5]. Conversely, a low 25(OH)D result should not automatically trigger high-dose replacement in a patient whose granulomatous disease may be generating active vitamin D independently of the kidney. Assessment of 1,25(OH)_2_D together with calcium and renal parameters can help identify patients in whom conventional replacement deserves caution [1,5].

Ultimately, the clinically meaningful endpoint is not simply normalization of a laboratory value. It is correction of clinically relevant vitamin D deficiency without provoking hypercalcemia or hypercalciuria. For patients with granulomatous disease, individualized assessment incorporating 25(OH)D, 1,25(OH)_2_D, calcium status, renal function, disease context, and the indication for treatment offers a more physiologically informed framework than a universal supplementation limit [1,5]. This is a clinical perspective rather than a proposed treatment guideline, and prospective studies are needed to determine whether metabolite-guided supplementation improves skeletal outcomes while reducing calcium-related harm.
